# The role of social support and sociocultural adjustment for international students' mental health

**DOI:** 10.1038/s41598-022-27123-9

**Published:** 2023-01-17

**Authors:** Jevgenij Razgulin, Gita Argustaitė-Zailskienė, Kastytis Šmigelskas

**Affiliations:** Department of Health Psychology, Faculty of Public Health, Medical Academy, Lithuanian University of Health Sciences, Tilžės G. 18, 47181 Kaunas, Lithuania

**Keywords:** Psychology, Signs and symptoms

## Abstract

The study aims to assess the role of social support, sociocultural adjustment, and other social and demographic factors in international students' mental health. In total, 193 international first-year students studying Health and Technology Sciences in Lithuania filled out a self-report questionnaire. The study revealed that overall 59% of international students had symptoms of depression and 36%—of anxiety. Students' well-being was sufficient in 56% of the cases. The regression analyses were conducted to test the role of sociocultural adjustment, social support, English reading skills, and the study field for mental health and well-being. The multivariate regression model revealed that sociocultural adjustment is a stronger predictor compared to social support for depressiveness (βs = 0.42), anxiety (βs = 0.30), psychosomatic symptoms (βs = − 0.24), and subjective health (βs = 0.16), though social support was a stronger independent predictor for well-being (βs = 0.37). Moreover, sociocultural adjustment and social support were stronger predictors than gender, while English reading skills and study field were non-significant indicators.

## Introduction

Worldwide, 5.67 million higher education students were studying abroad in 2018, and this number is growing every year^1^. Studying in another country can be a great opportunity, albeit difficult and challenging. Studies show that the challenges the international students face are bigger than those of local students^2^. A lot of difficulties may arise during foreign students' sociocultural adjustment to a new environment which could lead to the worsening of their psychological well-being and mental health. Some studies^3^ show that international students experience greater social isolation than the locals as they usually do not have strong social support in their new environment. A study by Han et al. reveals that because of the need to adapt to a new culture and a lack of social support, international students are classified as a group at risk, and they are more likely to have a variety of psychological problems^4^. However, sociocultural adjustment and social support are both modifiable factors. Therefore, it is important to understand what role do they play in the mental health of international students and which one is a stronger predictor of those indicators. This knowledge might help the universities to choose and implementing effective methods and programmes which would ensure better mental health of the international students.

This study aimed to assess the importance of sociocultural adjustment, social support, and other social and demographic factors for the students' mental health (depressiveness, anxiety, well-being, subjective health evaluation, and psychosomatic symptoms). Based on the previous literature it can be hypothesised that sociocultural adjustment, social support, and other social factors do affect the students' mental health.

Unfortunately, not many studies on these topics have been conducted in Eastern European countries, post-soviet countries or the countries where English or other popular languages do not dominate. Most studies with international students are done mainly in multicultural, mostly English-speaking countries^5^ and it might be problematic to extrapolate those results to countries with different sociocultural, and historical contexts, such as Post-soviet or other Eastern European countries. Studies about international students related to small-scale languages are also relatively scarce. It can be assumed that the issues raised above for countries with less popular languages are even more pressing, even if studies are conducted in a lingua franca. The findings of this study might enclose how international students adjust to the cultural environment where they can’t communicate well with people from the host culture due to the language and cultural barrier and which prevents them from participating in intercultural social activities and from engaging in the host society^6^. Problems international students are facing in Lithuania due to the language barrier might be similar to those where the local language differs from the study languages. For example, a study conducted with international students studying in Finland shows that international students experience more difficulties due to the language barrier than the locals since many teachers are not fluent in English. Also, sometimes organisational information is available only in the host country’s language, causing many difficulties in reaching study information or information about academic and social events^7^. Therefore, the language and cultural barrier might deepen the gap between international and local students.

These findings, which are conducted in a non-English speaking Eastern European country, could be used to formulate a more systematic approach, implementing more student support programs at the universities with similar sociocultural contexts and providing international students with better and more effective services in the future.

## Sociocultural adjustment

The ability of an individual to adapt to a new environment is referred to as sociocultural adjustment^8^. In the process of sociocultural adjustment, a person gains new skills and it covers some competencies needed to cope with different challenges of everyday life in a new cultural environment^9^. It is also shaped by learning about the culture as well as the acquisition of some social skills, among other things^10^. Sociocultural adjustment is a dynamic process that can lead to a good level of fit between a person and the environment^11^. However, if a person is lacking new skills which are needed and is functioning poorly in a new sociocultural environment, it could lead to the worsening of their mental health. This has been seen in studies on some relocation-related youth samples^12^ and could potentially be seen in international students as well. Older literature on expatriate experiences can also be consulted here; language, communication barriers, practical concerns such as housing, and other sociocultural obstacles have been found to be associated with stress, which could in turn affect mental health^13^. Overall, the previous studies reveal that sociocultural adjustment might have an important role in mental health of the international students.

## Social support

Sociocultural adjustment is closely related to social support, which is considered an important factor during the cross-cultural transition. International students often experience impaired social support due to losing the connection with their previous social support system. Developing a social network in a new country can be difficult for international students who risk social isolation, and that may impact their psychological well-being^6^. Hefner and Eisenberg^14^ found that international students are at a bigger risk of social isolation as well as a higher risk to experience mental health problems. Smith and Khawaja^15^ found that lack of language skills can cause some difficulties in everyday social interactions, possibly leading to a lack of social support. The situation could be even more difficult for international students who are studying in small countries where a local language is different from their study language. Some studies show that international students with better knowledge of English face fewer difficulties in their adjustment^16,17^, however, the inability to communicate well with people from the host culture prevents international students from participating in intercultural social activities and engaging in the host society^6^. This isolation might lead to a higher level of anxiety and depressiveness. International students might face similar problems in Lithuania because the local language differs from the language in which they study. The results of the previous studies show that social support and language skills might be important factors for the mental health of international students.

## Methods

### Sample

#### Participants

Our target population were first-year international study programme students. They were selected from four different universities in Kaunas city, Lithuania*.* An adjustment in a new culture and social support, as well as evaluate their role in international students' depressiveness, anxiety, well-being, subjective health, and psychosomatic symptom rate were evaluated. The study was carried out from February to March 2020. The survey was conducted in study rooms after lectures. The permission was obtained from the Kaunas Regional Ethics Committee for Biomedical Research (No. BE-2-8, 08-01-2020). Research was undertaken in accordance with relevant guidelines and regulations, and informed consent was obtained from all participants.

In total, 193 international students participated in this study, aged 21.2 ± 3.05 years, on average. The largest part of the sample was studying life sciences (Medicine, Odontology, and Veterinary Medicine), while others were studying technical sciences (Engineering and Information Technology). Students came from different countries: Armenia, Austria, Azerbaijan, Belarus, China, Cyprus, Ecuador, Egypt, Finland, France, Germany, India, Ireland, Israel, Kazakhstan, Lebanon, Malaysia, Malta, Morocco, Norway, Portugal, Russia, SAR, Saudi Arabia, Slovakia, Spain, Sweden, Switzerland, Syria, Tajikistan, Turkey, UK, Ukraine. The majority of the international students reported that their English skills were very good or that English was their mother tongue. The distribution of the sample by sociodemographic groups and the level of English skills are presented in Table 1.

**Table 1:** Main characteristics of study sample.

Demographics					
Gender	Number	Percent	By gender		
			Male (number)	Female (number)	
Male	117	60.6			
Female	76	39.4			

Study field	Study programme				
Life sciences, health sciences and, veterinary medicine	Medicine	82	42.5	37	45
	Odontology	26	13.5	19	7
	Veterinary medicine	23	11.9	4	19
	Physiotherapy	4	2.1	3	1
	Biotechnologies	3	1.6	2	1
Engineering and computing	Information Technology	16	8.3	15	1
	Mechatronics	10	5.2	10	0
	Aviation Engineering	9	4.7	8	1
	Electronic Engineering	5	2.6	5	0
	Civil Engineering	4	2.1	4	0
	Mechanical Engineering	4	2.1	4	0
	Materials Physics and Nanotechnology	3	1.6	3	0
	Chemical Engineering	1	0.5	1	0
	Chemical Technologies	1	0.5	0	1
	Not indicated	2	1.0	2	0

**Religion**					
Non-religious	43	22.3	23	20	
Christians	42	21.8	22	20	
Jews	42	21.8	25	17	
Muslims	38	19.7	27	11	
Hindus	14	7.3	11	3	
Others	7	3.6	5	2	
Not indicated	7	3.6	4	3	
**Region**					
European Economic Area (EEA)	70	36.3	26	44	
Israel	56	29.0	34	22	
India and Southeast Asia	27	14.0	21	6	
Post-Soviet countries except EEA	18	9.3	17	1	
Arab countries and Turkey	17	8.8	16	1	
Other	5	2.6	3	2	

English language skills					
Reading	Poor	6	3.1	5	1
	Good	41	21.2	22	19
	Very good	124	64.2	78	46
	Mother tongue	22	11.4	12	10
Writing	Poor	8	4.1	4	4
	Good	56	29.0	32	24
	Very good	110	57.0	70	40
	Mother tongue	19	9.8	11	8
Listening	Poor	3	1.6	2	1
	Good	40	20.7	22	18
	Very good	125	64.8	79	46
	Mother tongue	25	13.0	14	11
Speaking	Poor	4	2.1	2	2
	Good	64	33.2	38	26
	Very good	103	53.4	64	39
	Mother tongue	22	11.4	13	9

### Tools

Data were collected using a self-reported questionnaire. To assess sociocultural adjustment, the Sociocultural Adaptation Scale (SCAS) was used^18^. It has 41 items and can be modified according to sample characteristics; therefore, we only used the first 10 items as has been done previously in many cross-sectional studies and comparative analyses. Items are scored on a 5-point scale (from ‘no difficulty’ to ‘extreme difficulty'). A lower score indicates better sociocultural adjustment. The respondents must indicate the amount of difficulty they are experiencing in different areas such as understanding local perspectives and values, also communication and management of impersonal interactions (e.g., bureaucracy, authority) and/or awkward situations, however, difficulty is not explicitly framed in affective terms relating to anxiety, discomfort, and embarrassment. The examples of items could be “Making yourself understood”; “Getting used to the pace of life” and “Understanding jokes and humour”. In our study, the 10-item scale’s internal consistency was 0.81.

The Multidimensional Scale of Perceived Social Support^19^ was used to measure social support. The scale consists of 12 items grouped into three subscales of equal size—family, friends, and significant others. The items are rated on a 7-point Likert-type scale (from ‘very strongly disagree’ to ‘very strongly agree’). A higher score indicates better perceived social support. A score ranging from 1 to 2.9 could be considered low support; a score of 3 to 5—moderate support; a score from 5.1 to 7—high support.

Zimet et al. divides social support scale into three specific dimensions—significant others, the family and friends. Each dimension of the scales includes items which measure perceived availability of support for example: “My family is willing to help me make decisions”, and function of the support “I get the emotional help and support I need from my family”. In this study, the internal consistency of the scale was 0.92.

For measuring the level of depressiveness, the Center for Epidemiologic Studies Depression Scale Revised (CESD-R10)^20^ scale was used. It is a self-reported 10-item scale. The total score is calculated by summing the 10 items scores. A higher score indicates more symptoms of depression. The examples of items could be “I had trouble keeping my mind on what I was doing”; “I felt that everything I did was an effort”; “My sleep was restless”. A person scoring 10 or more is considered depressed. In our study, the scale’s internal consistency was 0.79.

To measure the risk of general anxiety disorder, the GAD-7 scale^21^ was used. This brief self-report scale consisting of 7 items. A higher score indicates higher level of anxiety. The examples of items could be “Feeling nervous, anxious or on edge”; “Having trouble relaxing” and “Feeling afraid, as if something awful might happen”. A score of 10 or greater represents a cut-off for identifying cases of GAD. Cut-off points of 5, 10, and 15 might be interpreted as representing mild, moderate, and severe levels of anxiety on the GAD-7. In our study, the scale’s internal consistency was 0.88.

For the measurement of current well-being, the WHO (Five) Well-Being Index (1998 version)^22^ scale was used. The WHO-5 is a self-report instrument with five statements scored concerning the past 2 weeks in a five-point scale (from ‘all of the time’ to ‘at no time’). A higher score indicates better well-being. The examples of items could be "I have felt cheerful and in good spirits”; “I have felt calm and relaxed” and “I have felt active and vigorous”. The total raw score, ranging from 0 to 25, is multiplied by 4 to give the final score, with 0 representing the worst and 100 representing the best imaginable well-being. In our study, the scale’s internal consistency was 0.86.

To measure the students' health status, the Self-rated Health Question^23^ was used. The question “How do you rate your health” is scored on a 5-point scale from ‘very good’ to ‘very bad’. A lower score indicates better health.

To measure the subjective health complaints, the HBSC Symptom Checklist (HBSC-SCL) was used. The scale measures different traits of psychosomatic complaints^24,25^. The HBSC-SCL is a non-clinical measure with 8 health complaint items: headache; abdominal pain; backache; feeling low; irritability or in a bad mood; feeling nervous; sleeping difficulties, and dizziness. Students were asked how often they experienced these symptoms over the last 6 months. The responses range from 'about every day' to 'rarely or never'. A lower score indicates more health complains. In our study, the scale’s internal consistency was 0.86.

### Data analysis

Statistical analyses were performed using IBM SPSS Statistics for Windows, Version 27 (IBM Corp., Armonk, N.Y., USA). Descriptive statistics were presented by arithmetic mean ± (SD) and by proportions (%). The Chi-square test was used to examine the differences between categorical variables. Spearman rank correlation coefficient was used to measure the strength and direction of associations between the ordinal variables. The univariate and multivariate linear regression was carried out to measure the role of social support and sociocultural adjustment for the mental health of international students. Cronbach's alpha coefficient was used to assess the internal consistency of SCAS, MSPSS, CESD-R10, GAD-7, and HBSC-SCL. The statistical significance level was set at 5%.

## Results

### Descriptives

Descriptive characteristics of the main variables are presented in Table 2. Skewness and kurtosis coefficients were used to test the normality of the distribution of data. According to these criteria, all variables are distributed normally except for social support.

**Table 2:** Distributions of the main indicators under study.

Indicator (scale)	Mean	SD	Median	Scale range	Skewness	Kurtosis
Social support (MSPSS)	5.6	1.26	5.8	1–7	− 0.83	1.38
Socio-cultural adjustment (SCAS)	1.9	0.64	1.8	1–5	0.56	− 0.47
Depressiveness (CES-D)	11.1	5.68	11.0	0–3	0.02	− 0.77
Anxiety (GAD-7)	8.3	5.6	8.0	0–3	0.52	− 0.49
Well-being (WHO-5)	13.3	5.36	13.0	0–5	− 0.20	− 0.73
Subjective Health (SH)	1.9	0.78	2.0	1–5	0.42	− 0.41
Psychosomatic (PS)	3.7	0.93	3.8	1–5	− 0.51	− 0.64

The majority of the sample reported their overall perceived social support as high (71.5%), while 24.7% reported moderate, and 3.8%—low social support. The mean score of social support was 1.9 (SD = 0.64) with 1 being the highest and 5 the lowest. 58.6% of the students (54.8% male, 64.5% female; χ^2^ = 1.77, p = 0.183) reported symptoms consistent with depression. 19.7% of the sample reported symptoms of anxiety, while 16.6% met the criteria for severe anxiety. The students' well-being was sufficient in 56.5% of the cases. Other descriptive statistics are shown in Table 2.

By gender, no differences in symptoms of depression, well-being, subjective health, and psychosomatic symptoms were observed (p > 0.05; Table 3). However, women reported more symptoms of anxiety than men (t = − 2.81, p = 0.005). Moreover, students claiming very good English reading skills as well as English being their mother tongue reported fewer depression symptoms, higher well-being, and better subjective health (p < 0.05). The respondents' study program was not associated with any of the abovementioned variables (p > 0.05).

**Table 3:** Mental health indicators by gender and studying-related factors.

Variable	Value	Depressiveness	Anxiety	Well-being	Subjective health	Psychosomatic symptoms
Gender	Male	10.52 ± 5.64	7.37 ± 5.64	13.81 ± 5.23	1.97 ± 0.82	3.83 ± 0.91
	Female	12.05 ± 5.64	9.64 ± 5.63	12.46 ± 5.49	1.92 ± 0.72	3.48 ± 0.91
	Difference	t = − 1.84, p = 0.068	t = − 2.81, p = 0.005*	t = 1.72, p = 0.87	t = 0.39, p = 0.699	t = 2.55, p = 0.12
Study programme	Life sciences, health sciences and, veterinary medicine	11.27 ± 5.74	8.62 ± 5.63	13.244 ± 5.37	1.93 ± 0.78	3.64 ± 0.94
	Engineering and computing	10.87 ± 5.53	7.38 ± 5.49	13.29 ± 5.40	2.02 ± 0.75	3.80 ± 0.89
	Difference	t = 0.441, p = 0.660	t = 1.404, p = 0.162	t = − 0.048, p = 0.962	t = − 0.737, p = 0.462	t = − 1.081, p = 0.281
English reading skills	Very good or mother tongue	10.60 ± 5.56	7.90 ± 5.59	13.81 ± 5.33	1.88 ± 0.77	3.74 ± 0.93
	Other	12.80 ± 5.76	9.40 ± 5.52	11.64 ± 5.16	2.15 ± 0.78	3.55 ± 0.92
	Difference	t = 2.32, p = 0.021*	t = 1.612, p = 0.109	t = − 2.45, p = 0.015*	t = 2.04, p = 0.043*	t = − 1.25, p = 0.212

*p < 0.05.

Women also expressed significantly lower sociocultural adaptation but significantly higher perceived social support (Table 4). By English reading skills and study profile (life sciences or technical sciences), the students did not differ in their sociocultural adaptation or perceived social support (p > 0.05).

**Table 4:** Sociocultural adjustment and social support by gender and studying-related factors.

Variable	Value	Sociocultural adjustment	Social support
Gender	Male	1.84 ± 0.62	5.39 ± 1.32
	Female	2.03 ± 0.66	6.04 ± 1.05
	Difference	t = − 2.02. p = 0.045*	t = − 3.605. p < 0.001*
Study programme	Life sciences, health sciences and, veterinary medicine	1.94 ± 0.67	5.87 ± 1.08
	Engineering and computing	1.86 ± 0.57	5.08 ± 1.50
	Difference	t = 0.75 p = 0.140	t = 4.07 p = 0.50
EN reading skills	Very good or mother tongue	1.84 ± 0.63	5.71 ± 1.19
	Other	2.04 ± 0.67	5.45 ± 1.46
	Difference	t = 1.56 p = 0.77	t = − 1.26 p = 0.246

*p < 0.05.

### Associations of perceived social support and sociocultural adjustment with student health

Based on correlations on social support, sociocultural adjustment with students health, well-being and age, it was revealed that, perceived social support was higher among students who did not exhibit psychosomatic symptoms or those of depressiveness/anxiety. Students who rated their well-being and subjective health higher, as well as older students, were also more likely to report better perceived social support. The associations between sociocultural adjustment and psychosomatic symptoms, depressiveness and anxiety, well-being, subjective health, psychosomatic symptoms and age followed the same pattern, except age which showed no correlation with sociocultural adjustment as can be seen in Table 5.

**Table 5:** Correlations between social, mental health and well-being indicators.

Variable	Social support		Sociocultural adjustment	
	r	p	r	p
Depressiveness	− 0.35	< 0.001*	0.52	< 0.001*
Anxiety	− 0.22	0.002*	0.38	< 0.001*
Well-being	0.38	< 0.001*	− 0.38	< 0.001*
Subjective health	0.18	0.010*	− 0.39	< 0.001*
Psychosomatic symptoms	− 0.18	0.012*	0.20	0.006*
Age	0.15	0.044*	− 0.11	0.138

*p < 0.05.

Higher perceived social support was associated with better well-being and subjective health as well as with a lower prevalence of anxiety, depressiveness, and psychosomatic symptoms (p < 0.05). Similar but stronger correlations were found regarding sociocultural adaptation and mental health measures (p < 0.05): lower adaptation was related to depression (r = 0.52), anxiety (r = 0.38), lower well-being (r = − 0.38), and worse subjective health (r = − 0.39).

### Linear regression analysis

Based on correlations on social support, sociocultural adjustment with students’ health, linear regression was carried out to measure the role of social support and sociocultural adjustment in the mental health of international students. Linear regression models were created to predict depressiveness, anxiety, well-being, subjective health, and psychosomatic symptoms based on social support and sociocultural adjustment; gender, study programme field, and English reading skills were also added to the models. The associations were assessed using univariate and multivariate approaches. We have measured results with β and β_s_. Finally, the strength of the links was evaluated by a standardized Beta (β_s_) in a multidimensional regression model (Table 6).

**Table 6:** The role of sociocultural adjustment and social support on mental health: linear regression analysis.

Variable	Value	Crude model					Multivariate model				
		Depressiveness	Anxiety	Well-being	Subjective health	Psychosomatic	Depressiveness	Anxiety	Well-being	Sunjective health	Psychosomatic
Social support	β	− 1.59	− 0.99	1.63	− 0.11	− 0.34	− 1.35	− 0.96	1.57	− 0.09	− 0.32
	βs	− 0.35	− 0.22	0.38	− 0.18	− 0.20	− 0.30	− 0.22	0.37	− 0.14	0.13
	p	< 0.001*	0.002*	< 0.001*	0.012*	0.005*	< 0.001*	0.003*	< 0.001*	0.078	0.013*
Sociocultural adjustment	β	4.56	3.30	− 3.13	0.24	− 1.04	3.74	2.62	− 2.17	0.19	0.85
	βs	0.52	0.38	− 0.38	0.20	− 0.32	0.42	0.30	− 0.26	0.16	− 0.24
	p	< 0.001*	< 0.001*	< 0.001*	0.006*	< 0.001*	< 0.001*	< 0.001*	< 0.001*	0.036*	< 0.001*
Gender	β	1.53	2.28	− 1.35	− 0.05	0.55	1.36	2.10	− 1.67	− 0.05	0.50
	βs	0.13	0.20	− 0.12	− 0.03	0.13	0.12	0.18	− 0.15	− 0.03	0.11
	p	0.068	0.005*	0.087	0.699	0.086	0.087	0.016*	0.037*	0.692	0.154
Study profile	β	− 0.36	− 1.13	− 0.04	0.07	− 0.30	− 0.75	− 0.80	0.58	− 0.05	− 0.25
	βs	− 0.03	− 0.09	0.00	0.04	− 0.64	− 0.06	− 0.06	0.05	− 0.03	− 0.05
	p	0.693	0.205	0.962	0.563	0.382	0.394	0.407	0.508	0.749	0.512
English reading skills	β	− 2.20	− 1.51	2.17	− 0.27	− 0.25	− 1.01	− 0.87	1.44	− 0.22	− 0.14
	βs	− 0.17	− 0.12	0.17	− 0.15	− 0.07	− 0.09	− 0.07	0.12	− 0.12	− 0.03
	p	0.021*	0.109	0.015*	0.043*	0.341	0.091	0.324	0.076	0.098	0.705
Model fit	R^2^						0.36	0.21	0.28	0.07	0.14
	p						< 0.001	< 0.001	< 0.001	0.014	< 0.001

*p < 0.05.

Univariate (crude) models between each independent and dependent variable showed that higher sociocultural adjustment and perceived social support were associated with lower depressiveness and anxiety, higher well-being, better subjective health, and fewer psychosomatic symptoms. Better English reading skills were associated with lower anxiety, higher well-being, and better subjective health. The female gender was also associated with higher anxiety. Notably, different study fields were not associated with any of the dependent variables.

The multivariate linear regression models were designed to establish the independent risk factors for the lower well-being of international students. Perceived social support, sociocultural adjustment, gender, study field, and English skills were among the independent factors. The results are detailed in Table 6. The multivariate model showed that sociocultural adjustment was a stronger predictor for depressiveness (β_s_ = 0.42), anxiety (β_s_ = 0.30), psychosomatic symptoms (β_s_ = − 0.24), and subjective health (β_s_ = 0.16), compared to social support (all p < 0.05). However, social support was a stronger independent predictor for well-being (β_s_ = 0.37, p < 0.05). Both sociocultural adjustment and social support were stronger predictors for the students’ mental health than gender, while the female gender was associated with more anxiety (β_s_ = 0.18), lower well-being (β_s_ = − 0.15), but not with depressiveness, psychosomatic symptoms, or subjective health (p > 0.05). In contrast, study field and English skills were not independent predictors for international students’ mental health. Of note, multivariate regression estimates did not differ essentially from that of univariate regression.

All multivariate models were statistically significant and fit the data except the model predicting subjective health (R^2^ = 0.07, p = 0.014).

## Discussion and conclusions

This study aimed to assess the importance of social support, sociocultural adaptation, and other social and demographic factors for student mental health in terms of depressiveness, anxiety, well-being, subjective health evaluation, and psychosomatic symptoms. The results revealed that the prevalence of depressiveness and anxiety was quite high among international students and that sociocultural adjustment played one of the main roles in predicting the students' mental health. Social support was another slightly weaker but still significant predictor compared to sociocultural adjustment.

This study revealed that 59% of the international students had symptoms consistent with depression, 20% of the students had moderate symptoms of anxiety, and 17% of students met the criteria for severe anxiety. The students’ well-being was sufficient in 56% of the cases. The number of international students in Lithuania with symptoms of anxiety and depressiveness was quite high. Akhtar et al.^26^ who conducted a study with international medical students in Germany where 26% of these students had symptoms of depression and 49% of those students had symptoms of anxiety. A study by Han et al.^4^ among Chinese international students in the USA revealed that 45% of them had symptoms of depression and 29% of them had symptoms of anxiety. The prevalence rate of depression in Indian students in Kazakhstan was 33%, the rate of anxiety was 21%^27^. Studies conducted during COVID-19 pandemic also show high prevalence of depression and anxiety among students. A study by Ochnik, D., conducted in 9 countries (Poland, Slovenia, Check Republic, Ukraine, Russia, Germany, Turkey, Israel and Colombia) revealed that in total 40% of these students suffered from the moderate or severe symptoms depression, with the highest prevalence in Turkey (62%) and lowest in Check Republic (21%). Moderate or severe anxiety symptoms were observed among 30% of those students from 9 countries, with highest prevalence in Turkey (51%) and lowest in Germany (5%) and Check Republic (13%)^28^.

The results of these studies show that similarly to our study results, university students have a quite high prevalence of depression and anxiety across different countries.

The multivariate model where sociocultural adjustment and social support were the independent variables and mental health indicators are the dependent variables revealed that sociocultural adjustment is a stronger predictor for such student mental health indicators as depressiveness, anxiety, psychosomatic symptoms, and subjective health, compared to social support. In contrast, social support was a stronger independent predictor for well-being. Moreover, sociocultural adjustment and social support were stronger predictors for student mental health than gender. The female gender was linked with more anxiety and lower well-being; however, gender was not associated with depressiveness, psychosomatic symptoms, or subjective health. Study by Werner et al. also revealed that female students reported more anxiety, however that study contradict to our results as female students do not show significant difference in depression or somatic complaints in our study^29^. The multivariate model in our study also showed that even though English reading skills were linked with depressiveness and well-being and subjective health in the univariate regression model, this variable does not predict those indicators any longer in a multivariate regression model which means that English reading skills are not an independent variable and the link with the international student mental health might be caused by the other independent variables. Also, the study field did not predict international students’ mental health.

The findings of Swami et al.^30^ similarly show that the importance of the role of sociocultural adjustment in mental health indicators is high and suggest that sociocultural adjustment is associated with subjective health. Social support was also found to be a significant predictor of mental health in a study by Atri et al.^31^. It reveals that the belonging aspect of social support is very important for one's mental health. If a person has higher emotional social support and has somebody with whom they might express and ventilate their emotions then their chances to have better mental health increase.

## Limitations

The sample size was not large due to a limited number of first-year international students in Lithuanian universities. Also, some of the students were absent at the moment of the study. The study was done just before the lockdown due to COVID-19, thus it may have been already affecting the mental state of the international students. The questionnaire was presented in English, not in the students native languages and it could have made an impact on the understanding of some statements. The sample of our study was very diverse in their nationalities and ethnic background, which could limit the possibility of a more accurate comparison of these studies. In some cultures, mental health is still a taboo subject and it is not acceptable to talk about mental health problems openly. Therefore, it might affect some answers of participants of this study. Such factors as acculturation stress, perceived discrimination, or acculturation strategies might also be important predictors of the mental health of international students but they were not included in this study. These factors should be considered in future studies.

## Conclusion

This study revealed the importance of sociocultural adjustment, social support, and other social and demographic factors for students’ mental health. It also showed that sociocultural adjustment is the strongest of those predictors. Moreover, this study revealed that mental health problems are quite common among international students, therefore they should be taken into consideration by the university authorities and professionals. Furthermore, sociocultural adjustment and social support are modifiable factors, and many actions could be taken by involving the university community in the building of a supportive and healthier environment. Consequently, a friendly and open environment can help international students to adjust and feel better in a new place. Student support programmes, such as a mentoring programme, should be implemented, as they can help students to get better social support, stay healthy, and enjoy a higher well-being. Moreover, there should be courses and other activities for the international students which can help them to understand the host culture better and to prepare for the new challenges.

## Data Availability

The datasets used and analysed during the current study is available from the corresponding author on reasonable request.
